# Herpes Zoster in Solid Organ Transplantation: Incidence and Risk Factors

**DOI:** 10.3389/fimmu.2021.645718

**Published:** 2021-03-18

**Authors:** Marcia M. L. Kho, Stefan Roest, Dominique M. Bovée, Herold J. Metselaar, Rogier A. S. Hoek, Annemiek A. van der Eijk, Olivier C. Manintveld, Joke I. Roodnat, Nicole M. van Besouw

**Affiliations:** ^1^ Department of Internal Medicine-Nephrology and Transplantation, Erasmus MC, University Medical Centre Rotterdam, Rotterdam, Netherlands; ^2^ Erasmus MC Transplant Institute, University Medical Center Rotterdam, Rotterdam, Netherlands; ^3^ Department of Cardiology, Thorax Center, Erasmus MC, University Medical Centre Rotterdam, Rotterdam, Netherlands; ^4^ Department of Gastroenterology and Hepatology, Erasmus MC, University Medical Centre Rotterdam, Rotterdam, Netherlands; ^5^ Department of Respiratory Medicine, Erasmus MC, University Medical Centre Rotterdam, Rotterdam, Netherlands; ^6^ Department of Viroscience, Erasmus MC, University Medical Centre Rotterdam, Rotterdam, Netherlands

**Keywords:** herpes zoster, incidence, organ transplantation, risk factors, varicella zoster virus

## Abstract

**Background:**

Studies on herpes zoster (HZ) incidence in solid organ transplant (SOT) recipients report widely varying numbers. We investigated HZ incidence, severity, and risk factors in recipients of four different SOTs, with a follow-up time of 6–14 years.

**Methods:**

Records of 1,033 transplant recipients after first heart (HTx: n = 211), lung (LuTx: n = 121), liver (LiTx: n = 258) and kidney (KTx: n = 443) transplantation between 2000 and 2014 were analyzed for VZV-PCR, clinical signs of HZ, and complications.

**Results:**

HZ was diagnosed in 108 of 1,033 patients (10.5%): 36 HTx, 17 LuTx, 15 LiTx, and 40 KTx recipients. Overall HZ incidence rate after HTx (30.7 cases/1,000 person–years (PY)), LuTx (38.8 cases/1,000 PY), LiTx (22.7 cases/1,000 PY) and KTx (14.5 cases/1,000 PY) was significantly higher than in the general 50–70 year population. Multivariable analysis demonstrated age ≥50 years at transplantation (p = 0.038, RR 1.536), type of organ transplant (overall p = 0.002; LuTx p = 0.393; RR 1.314; LiTx p = 0.011, RR 0.444; KTx p = 0.034, RR 0.575), CMV prophylaxis (p = 0.043, RR 0.631) and type of anti-rejection therapy (overall p = 0.020; methylprednisolone p = 0.008, RR 0.475; r-ATG p = 0.64, RR1.194) as significant risk factors. Complications occurred in 33 of 108 (31%) patients (39% of HTx, 47% of LuTx, 20% of LiTx, 20% of KTx): post-herpetic neuralgia, disseminated disease, and cranial nerve involvement.

**Conclusion:**

HZ incidence and severity in SOT recipients are most pronounced after heart and lung transplantation, in older patients, and when CMV prophylaxis is lacking.

## Introduction

Herpes zoster (shingles, HZ) is caused by reactivation of the varicella zoster virus (VZV). After primary infection, the virus establishes lifelong latency in the dorsal root neural ganglia (1). Virus reactivation occurs when the immune system is suppressed. Increased HZ incidence, attributed to a decline in immunity, is observed in elderly people and in patients using immunosuppressive medication (2–10). The latter certainly applies to solid organ transplant recipients.

Both HZ incidence and HZ related complications occur more frequently and with higher severity in solid organ transplant recipients compared to the general population (11). Severe complications are dissemination in more than three dermatomas, involvement of cranial nerves or internal organs and post-herpetic neuralgia (PHN). PHN may lead to considerable morbidity and loss of quality of life (12). Due to the use of different definitions of PHN, the reported incidence ranges from 10 to 50% of herpes zoster cases (13). In the Netherlands, overall annual HZ incidence is 3.2 cases per 1,000 person–years (PY), comparable to other West-European countries (3). HZ incidence increases with age up to 10 cases/1,000 PY (3) in people older than 80 years. In solid organ recipients however, HZ incidence has been reported to be two- to five-fold higher than in the general 80 years old population (14–18).

Studies on HZ incidence and complications in multiple solid organ transplant (SOT) recipients report widely varying numbers. In North America and Asia HZ incidence ranges from 18/1,000 PY in liver to 55/1,000 PY in lung transplant recipients (14–21). Whereas in Europe, kidney transplant studies show crude incidences of 1–8% (22, 23) and overall incidences of 20/1,000 PY (24), and one study of multiple SOT recipients reports an incidence of 12/1,000 PY (25). Therefore, we assessed crude and overall incidence and complications of HZ after heart, lung, liver, and kidney transplantation in our center by retrospective analysis of the medical files of adult recipients. Furthermore, we performed a detailed analysis of risk factors for developing HZ.

## Patients and Methods

**Figure 1:**
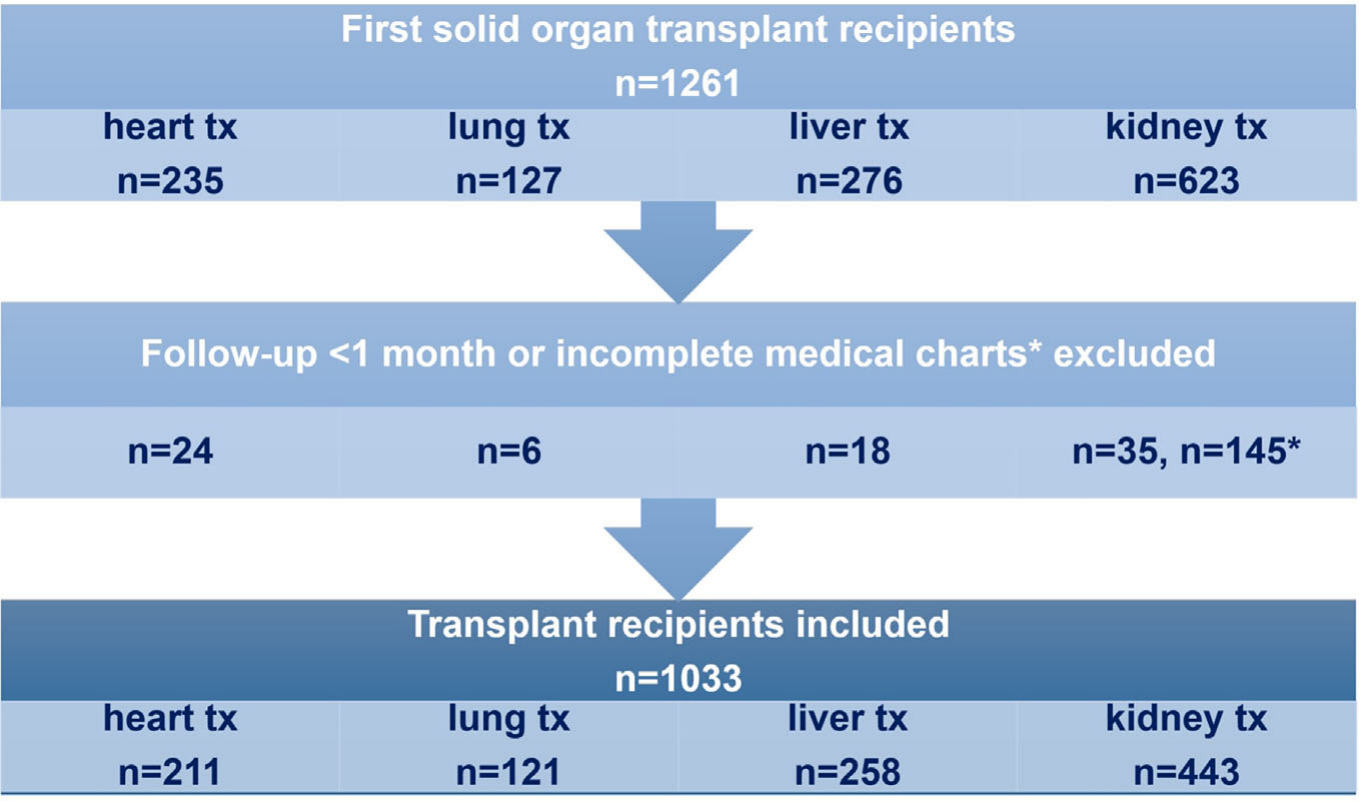
Adult recipients of a first solid organ transplant with a minimum follow-up of 1 month and complete medical charts were included in the analysis. tx= transplantation.

Medical records of adult heart (HTx), lung (LuTx), liver (LiTx), and kidney (KTx) transplant recipients in our transplant center between 2000 and 2014 were reviewed. Permission to extract data from the hospital (pharmacy) records was granted by the local medical ethical commission: MEC-2018-1574. Because not all KTx recipients visit our outpatient clinic in case of infection, we performed an inquiry by letter and phone calls. Patients who died or lost their graft within one month after a first organ transplantation were excluded from our analysis. KTx recipients whose medical records were incomplete, mostly due to referral to another hospital, and who did not respond to our inquiries, were excluded (**Figure 1**).

Demographic and clinical information was extracted from the medical records and included the following information: transplantation date, gender, date of birth, date of death or graft failure (if applicable), duration of follow-up, induction therapy, maintenance immunosuppressive regimen, use of methylprednisolone or rabbit Anti-Thymocyte Globulin (r-ATG) as anti-rejection therapy, cytomegalovirus (CMV) prophylaxis regimen, serum CMV-PCR results post-transplantation, patient CMV serologic status pre-transplantation, donor CMV serologic status, patient VZV serologic status (IgG positive or negative) pre-transplantation, first episode of HZ, location and number of dermatomas affected by HZ, internal organ and cranial nerve involvement, VZV-PCR results, therapy used to treat HZ, and occurrence of PHN. Primary varicella zoster infections were not included in our analysis.

Localized HZ was defined as presentation of vesicles in one or two adjacent dermatomas, whereas involvement of three or more, or two not adjacent dermatomas was considered as disseminated HZ (11). Cranial nerve involvement was scored separately. Post-herpetic neuralgia (PHN) was defined as pain in the affected dermatomas, persisting at least 3 months after onset of the skin lesions and requiring treatment with opioid analgesics, tricyclic antidepressants, gabapentin, or pregabalin (26). Infections were mostly confirmed by VZV-PCR on the blood and/or blister samples; however, in obvious cases HZ was diagnosed on clinical presentation only.

### Statistical Analysis

Analyses were performed in SPSS, version 25, 2017. HZ incidence was expressed as percentage of the total number of patients (crude incidence) and as cases per 1,000 person–years (overall incidence rate). Age at transplantation in all patients with and without HZ was compared by one-way ANOVA. Age at transplantation in patients with positive and negative VZV IgG before transplantation was also compared by one-way ANOVA. Correlation between time to HZ onset and age at transplantation was analyzed with Spearman’s correlation test. Univariable and multivariable Cox proportional hazards analysis using backward elimination was used to analyze the effect of multiple variables on HZ incidence. Univariable Cox proportional hazards analysis was used to analyze the effect of the type of organ transplant on complicated HZ incidence. Cases with missing values were excluded from Cox proportional hazards analyses.

## Results

### Patient Characteristics

In total, 1,261 patients received a first transplant, of which 235 HTx (Tx period January 2000–July 2014), 127 LuTx (Tx period April 2002–March 2014), 276 LTx (Tx period January 2008–July 2014), and 623 KTx (Tx period January 2003–January 2009). Of the 588 KTx patients with a follow-up of more than 1 month, 145 had incomplete medical charts, due to referral to another hospital and/or no response to our inquiry, resulting in 443 patients in the KTx group and a total of 1,033 patients (**Figure 1**). Maximum follow-up time was 14 years in HTx, 12 years in LuTx, 6 years in LiTx and 10 years in KTx recipients. Mean follow-up time was 5.5 years in HTx, 3.6 years in LuTx, 2.6 years in LiTx, and 6.0 years in KTx. During follow-up 26 (12%) HTx, 21 (17%) LuTx, 29 (11%) LiTx, and 14 (3%) KTx recipients died and 12 (5%) LiTx and 72 (16%) KTx recipients lost their graft.

The characteristics of the organ transplant recipients are shown in **Table 1**. In the LuTx group about 54% was male, whereas in the other groups the percentage of males varied between 62 and 68%. The standard immunosuppressive medication regimens at time of transplantation in each group are shown in **Table 1**. There are some differences in immunosuppressive medication between the transplant groups: all HTx recipients received r-ATG as induction therapy. After LiTx, patients received tacrolimus monotherapy as maintenance immunosuppressive therapy from 6 months post-transplantation. HTx and LuTx recipients received an increased dose of maintenance immunosuppression as compared to LiTx and KTx recipients (triple therapy including low dose prednisolone and higher tacrolimus target concentrations).

**Table 1:** Transplant recipients’ characteristics.

Organ transplant	Heart	Lung	Liver	Kidney	Overall
**Recipients in analysis**	211	121	258	443	1033
**Gender (M/F)**	143/68	65/56	169/89	274/169	649/384
**Median age at Tx (range)**	51 (18–72)	54 (19–66)	53 (18–69)	51 (18–77)	52 (18–77)
**Age ≥ 50 y at Tx (%)**	54%	66%	58%	53%	56%
**Pre-Tx VZV-IgG pos/neg/unknown**	194/11/6	108/5/8	239/7/12	419/15/9	960/38/35
**Induction therapy**	r-ATG211 (100%)	Basiliximab 121 (100%)	Basiliximab258 (100%)	2006–2008: rATG in DCD: 44 (10%) Rituximab in ABO-I: 18 (4%) No induction: 381 (86%)	
**Standard maintenance immunosuppression > 6 months**	Tacrolimus + Mycophenolate Mofetil + Prednisolone	Tacrolimus + Mycophenolate Mofetil + Prednisolone	Tacrolimus	Tacrolimus + Mycophenolate Mofetil	
**Tacrolimus target trough levels (ug/l)** **> 6 months**	<12 mo: 9–15 >12 mo: 5–9	<7 mo: 10–15 >7 mo: 7–10	5–8	5–8	
**r-ATG anti-rejection therapy (pts)**	15 (7%)	0	1 (0.4%)	32 (7%)	48 (5%)
**Methylprednisolone anti-rejection therapy (pts)**	72 (34%)	42 (35%)	30 (12%)	102 (23%)	246 (24%)
**CMV serostatus pre-transplant**					
** D−/R−** ** D−/R+** ** D+/R−** ** D+/R+** ** unknown**	36 (17%) 71 (34%) 59 (28%) 45 (21%) 0	30 (25%) 37 (31%) 23 (19%) 30 (25%) 1 (1%)	44 (17%) 80 (31%) 45 (17%) 89 (35%) 0	96 (22%) 102 (23%) 82 (19%) 154 (35%) 9 (2%)	206 (20%) 290 (28%) 209 (20%) 318 (31%) 10 (1%)
**CMV prophylaxis**					
** Yes** ** No** ** unknown**	59^a^ (28%) 152 (72%) 0	91 (75%) 30 (25%) 0	45 (17%) 213 (83%) 0	340 (77%) 99 (22%) 4 (1%)	535 (52%) 494 (48%) 4 (1%)

Tx, transplantation; M, male; F, female; D, donor; R, recipient; pts, patients; VZV-IgG, Varicella Zoster Virus immunoglobulin G; r-ATG, rabbit anti-thymocyte globulin; Basiliximab, InterLeukin2-receptor blocker; Rituximab, anti-CD20 antibody; DCD, donation after cardiac death. ^a^: five patients received immunoglobulin CMV prophylaxis.

In general, valganciclovir was used as CMV prophylaxis. In the LuTx and KTx groups, all except CMV donor negative/recipient negative combinations received prophylaxis. In the HTx and LiTx groups only CMV donor positive/recipient negative combinations received prophylaxis. In the HTx group up to 2003, prophylactic anti-CMV immunoglobulin was given in the first 6 weeks after transplantation. Since 2003, valganciclovir was given during the first 6 months after transplantation. In the LuTx group, duration of CMV prophylaxis was extended during the study period: up to 2012 valganciclovir was given during the first 3 months, since 2012 prophylaxis was extended to 6 months after transplantation. In the KTx and LiTx groups, valganciclovir was given during the first 3 months.

Most of the methylprednisolone treated rejections occurred early after transplantation, during the standard CMV prophylaxis period: 59/72 (82%) in HTx, 25/42 (60%) in LuTx, 14/30 (47%) in LiTx, 65/102 (64%) in KTx. CMV prophylaxis was not extended after methylprednisolone anti-rejection treatment.

### VZV-Seroprevalence

In the Netherlands, no routine VZV vaccination program exists, neither primary vaccination for the general population nor booster vaccination for senior adults. VZV is endemic in the Dutch population, seroprevalence of VZV antibodies amounts to 95% (27). Of our transplant candidates 3.7% (38/1033) was VZV seronegative prior to transplantation (**Table 1**).

Age at transplantation was significantly higher in patients who were VZV IgG seropositive compared to seronegative before transplantation [50.4 (17.5–77.8) *vs*. 45.7 (19.7–75.2), p=0.024].

Two KTx patients, one LuTx patient, and two HTx patients suffered from primary VZV infection, 1.2–8 years after transplantation. Although some had severe complications, none of them died.

Pre-transplantation vaccination of VZV seronegative patients was introduced only in the kidney transplant group [two doses of Provarivax (Merck Sharp & Dohme B.V., Haarlem) (28)]. Four of 15 VZV-seronegative kidney transplant candidates were vaccinated before transplantation with Provarivax. One of these vaccinated transplant recipients developed HZ during follow-up, at 2.7 years post-transplantation. This patient had non-complicated HZ and recovered without sequelae.

Booster vaccination of seropositive patients was not performed in any of the groups.

### Incidence and Severity of Herpes Zoster

To compare the incidence of HZ in our center with the incidence reported by other authors, we analyzed both crude and overall incidences. Only the first HZ episode after transplantation was analyzed. Primary VZV infections were not included in the analysis.

The crude HZ incidence was 36/211 (17.1%) in the heart, 17/121 (14.0%) in the lung, 15/258 (5.8%) in the liver, and 40/443 (9.2%) in the kidney transplant recipients (**Table 2**).

**Table 2:** Transplant recipients with herpes zoster.

Organ transplant	Heart	Lung	Liver	Kidney	Overall
**Herpes Zoster** ** Cases/recipients**	36/211 (17.1%)	17/121 (14.0%)	15/258 (5.8%)	40/443 (9.2%)	108/1033 (10.5%)
**Herpes Zoster** ** Cases/1,000 PY**	30.7	38.8	22.7	14.5	22.1
**Gender (M / F)**	27/9	9/8	11/4	25/15	72/36
**Median age at Tx (range)**	54 (22–67)	60 (35–67)	52 (22–60)	53 (28–77)	53 (21–72)
**HZ onset post-Tx** **Median years (range)**	2.0 (0.04−10.8)	1.4 (0.08−3.8)	0.5 (0.3−4.5)	1.8 (0.04−8.9)	1.2 (0.04−10.8)
**Initial HZ treatment**					
** Oral** ** Intravenous** ** No treatment**	32 (89%) 4 (11%) 0	9 (53%) 7 (41%) 1 (6%)	12 (80%) 3 (20%) 0	37 (92%) 3 (8%) 0	90 (83%) 17 (16%) 1 (1%)
**CMV-IgG pre-Tx**					
** D−/R−** ** D−/R+** ** D+/R−** ** D+/R+** ** unknown**	5 (14%) 19 (53%) 7 (19%) 5 (14%) 0	5 (29%) 7 (41%) 3 (18%) 2 (12%) 0	3 (20%) 5 (33%) 1 (7%) 6 (40%) 0	10 (25%) 7 (18%) 6 (15%) 16 (40%) 1 ( 3%)	23 (21%) 38 (35%) 17 (16%) 29 (27%) 1 (1%)
**CMV prophylaxis**					
** Yes** ** No** ** unknown**	7 (19%) 29 (81%) 0	12 (71%) 5 (8%) 0	1 (7%) 14 (23%) 0	28 (70%) 11 (28%) 1 (3%)	48 (44%) 59 (54%) 1 (1%)
**Induction therapy**	r-ATG 36 (100%)	Basiliximab 17 (100%)	Basiliximab 15 (100%)	2006–2008: rATG in DCD: 2 (5%) Rituximab in ABO-I: 3 (7.5%) No induction: 35 (87.5%)	r-ATG 38 (35%) Basiliximab 32 (30%) Rituximab 3 (3%) No 35 (32%)
**prior r-ATG anti-rejection therapy**	3 (8%)^a^	0	0	5 (13%)^a^	8 (7%)
**prior methylprednisolone anti-rejection therapy**	3 (8%)	5 (29%)^b^	3 (20%)^a^	5 (13%)	16 (15%)

T, transplantation; VZV-IgG, Varicella Zoster Virus immunoglobulin G; PY, person–years. M, male; F, female; D, donor; R, recipient; rATG, rabbit anti-thymoglobulin. ^a^: 1 complicated HZ case. ^b^: 3 complicated HZ cases.

**Figure 2:**
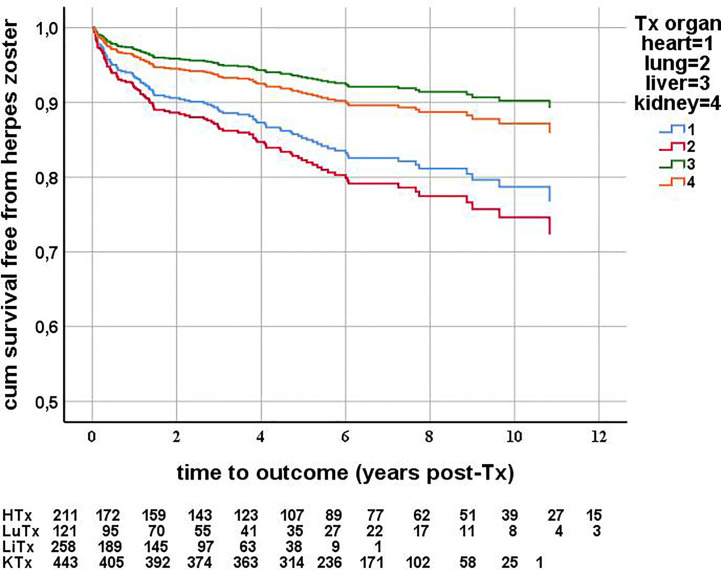
Herpes zoster free survival after solid organ transplantation, censored for death and graft failure. Cox proportional hazards, multivariable analysis. First episodes of herpes zoster were counted as event. Herpes zoster incidence is significantly higher in heart recipients compared to liver (p = 0.011) and kidney (p = 0.034) transplant recipients. For each organ type, the number of patients at risk at each year after transplantation is described below the graph.

The overall HZ incidence is shown in **Table 2** as the number of HZ cases per 1,000 person–years (PY), meaning the years at risk of HZ after transplantation. The HZ incidence was significantly higher after HTx (30.7 cases/1,000 PY) compared to after LiTx (22.7 cases/1,000 PY) and after KTx (14.5 cases/1,000 PY) (Cox proportional hazards, p < 0.001 in LiTx *vs.* HTx, p = 0.003 in KTx *vs.* HTx). HZ incidence after LuTx (38.8 cases/1,000 PY) was comparable after HTx (Cox proportional hazards, p = 0.907) (**Tables 2** and **4**, **Figure 2**).

The median time to the onset of HZ was 2.0 years after HTx, 1.4 years after LuTx, 0.5 years after LiTx, and 1.8 years after KTx (**Table 2**).

More than 80% of HZ episodes were treated with oral valacyclovir (**Table 2**). LuTx recipients more often received intravenous acyclovir treatment compared to recipients of other organs.

Complicated HZ incidence did not significantly differ between the four organ transplant groups (Cox proportional hazards, p = 0.156) (**Table 3**). The incidences of PHN, disseminated HZ, and cranial nerve involvement are shown in **Table 3**. One LuTx patient and two KTx recipients died due to disseminated HZ with secondary bacterial infection and encephalitis. Of the patients who had HZ after treatment for acute rejection, six had complicated HZ. Four patients (three LuTx, one LiTx) had received methylprednisolone, one HTx and one KTx patient received r-ATG (**Table 2**).

**Table 3:** Herpes zoster complications.

Organ transplant	Heart	Lung	Liver	Kidney	Overall
**Complicated/Total**	14/36 (39%)	8 /17 (47%)	3/15 (20%)	8/40 (20%)	33/108 (31%)
** RR (95% CI) p-value**	Reference	2.147 (0.831–5.550) 0.115	1.166 (0.314–4.326) 0.818	0.671 (0.269–1.670) 0.391	0.156
**Post Herpetic Neuralgia**	7 (19%)	3 (18%)	1 (7%)	0	11 (33%)
**Disseminated disease**	2 (6%)	5 (29%)	2 (13%)	7 (18%)	16 (48%)
**Cranial nerve involvement**	5 (14%)	1 (6%)	0	1 (3%)	7 (21%)
**Deceased due to complicated HZ**	0	1 (6%)	0	2 (5%)	3 (9%)

### Risk Factors for Herpes Zoster

We analyzed the risk factors for the development of HZ. Age at transplantation was not different between patients who did or did not develop HZ, neither in the entire group [51.7 (21.1–72.8) *vs*. 49.9 (17.5–77.8), p = 0.17), nor in the HTx, LuTx, LiTx, and KTx groups separately.

No correlation was found between the time to the onset of HZ after transplantation and age at transplantation (r_s_ = 0.009, p = 0.93).

As HZ incidence significantly increases in the general population above 50 years (3), we added age categories ≥50 years and ≥60 years at transplantation as dichotomous variables. We studied the effect of the following variables on the risk of developing HZ: gender, age at transplantation, age ≥50 years at transplantation, age ≥60 years at transplantation, type of organ transplant, use of methylprednisolone or r-ATG anti-rejection therapy, induction therapy agent (no induction, basiliximab, rATG, or rituximab), use of CMV prophylaxis (in all patients and in CMV seropositive recipients only), duration of CMV prophylaxis (none, valganciclovir 3 months, and ≥6 months), occurrence of CMV viremia (serum CMV-PCR >1000 IU/ml) and pre-transplant VZV-IgG (positive of negative) (**Tables 4** and **5**).

**Table 4:** Risk factors for herpes zoster, univariable analysis.

Variable (reference)	Cox proportional hazards Univariable analysis	
	RR (95% CI)	p-value
**Gender (male)**	0.834 (0.559–1.245)	0.374
**Age (continuous)**	1.012 (0.997–1.028)	0.125
**Age ≥50 years**	1.672 (1.120–2.495)	0.012
**Age ≥60 years**	1.355 (0.889–2.066)	0.158
**Organ transplant (Heart)**		0.003
** Lung** ** Liver** ** Kidney**	1.035 (0.579–1.850) 0.300 (0.159–0.565) 0.482 (0.297–0.783)	0.907 <0.001 0.003
**VZV IgG pre-transplant (negative)**	2.370 (0.585–9.611)	0.227
**Valganciclovir CMV prophylaxis (no)**	0.629 (0.430–0.922)	0.017
**CMV prophylaxis (no)**		0.041
** Valganciclovir 3 months** ** Valganciclovir >6 months**	0.603 (0.404–0.900) 0.669 (0.289–1.547)	0.013 0.347
**CMV prophylaxis (no)**		0.086
** Valganciclovir 3 months** ** Valganciclovir 6 months** ** Valganciclovir 9 months**	0.603 (0.405–0.900) 0.752 (0.302–1.872) 0.430 (0.059–3.115)	0.013 0.540 0.403
**Valganciclovir prophylaxis CMV R+ (no)****^a^**	0.550 (0.337–0.898)	0.017
**CMV-PCR >1,000 IU/ml**	1.012 (0.627–1.634)	0.960
**Induction therapy (no)**	1.676 (1.114–2.522)	0.013
**Induction therapy (no)**		0.035
** Basiliximab or Rituximab** ** rATG**	1.537 (0.952–2.482) 1.818 (1.144–2.887)	0.078 0.011
**Induction therapy (no)**		0.059
** Basiliximab** ** rATG** ** Rituximab**	1.484 (0.909–2.423) 1.817 (1.144–2.886) 2.432 (0.746–7.928)	0.114 0.011 0.140
**Anti-rejection therapy (no)** ** Methylprednisolone** ** r-ATG**	0.531 (0.311–0.906) 1.301 (0.629–2.691)	0.042 0.020 0.477
**r-ATG anti-rejection therapy (no)**	1.481 (0.720–3.050)	0.286

VZV IgG, varicella zoster virus specific immunoglobulin G; CMV, cytomegalovirus; r-ATG, rabbit anti-thymocyte globulin; CMV R+, CMV seropositive recipient.

a CMV seropositive recipients without Valganciclovir prophylaxis were compared to CMV seropositive recipients with Valganciclovir prophylaxis.

**Table 5:** Risk factors for herpes zoster, multivariable analysis.

Variable (reference)	Cox proportional hazards Multivariable analysis	
	RR (95% CI)	p-value
**Age (≥50 years)**	1.536 (1.023–2.304)	0.038
**Organ transplant (Heart)**		0.002
**Lung** **Liver** **Kidney**	1.314 (0.703–2.455) 0.444 (0.372–0.833) 0.575 (0.345–0.959)	0.393 0.011 0.034
**CMV prophylaxis (no)**	0.631 (0.404–0.986)	0.043
**Anti-rejection therapy (no)**		0.020
**Methylprednisolone** **r-ATG**	0.475 (0.275–0.821) 1.194 (0.566–2.518)	0.008 0.641

CMV, cytomegalovirus; r-ATG, rabbit anti-thymocyte globulin.

In univariable Cox regression analysis, age ≥50 years at transplantation, type of organ transplant, use of CMV prophylaxis, duration of CMV prophylaxis, use of induction therapy, type of induction therapy, and type of anti-rejection therapy significantly influenced the risk of developing HZ (**Table 4**).

In multivariable Cox regression analysis, successively including all the above mentioned variables, age ≥50 years at transplantation, type of organ transplant, use of CMV prophylaxis, and type of anti-rejection therapy were the variables significantly influencing the risk of developing HZ (**Table 5**). Patients ≥50 years of age had a significantly increased risk of developing HZ compared to younger patients (p = 0.038, RR = 1.536, CI = 1.023–2.304). Compared to HTx (reference variable) the risk of HZ after LuTx was not different. The risk of developing HZ after LiTx (p = 0.011, RR = 0.444, CI = 0.237–0.833) and after KTx (p = 0.034, RR = 0.575, CI = 0.345–0.959) was significantly lower than after HTx (**Figure 2**, **Table 5**). Use of CMV prophylaxis significantly diminished HZ risk (p = 0.043, RR = 0.631, CI = 0.40–0.986). The risk of developing HZ was significantly lower in patients who were treated for acute rejection with methylprednisolone, compared to those without acute rejection treatment (p = 0.008, RR = 0.475, CI = 0.275–0.821). In the multivariable Cox regression model, no interaction was found between type of organ transplant and either age ≥50 years, use of CMV prophylaxis, or type of anti-rejection therapy.

In addition, we performed a Cox regression analysis of the effect of the above mentioned variables on the risk of developing HZ in all organ transplant subgroups. In the LuTx and LiTx groups, none of the variables significantly influenced the incidence of HZ. In univariable analysis in the HTx group (n = 211 with 36 HZ cases) we found two significant risk factors for development of HZ: any anti-rejection therapy (p = 0.002, RR 0.253 (0.105–0.610) and type of anti-rejection therapy (overall p = 0.009; methylprednisolone p = 0.003, RR 0.165 (0.050–0.542); rATG p = 0.311, RR 0.541 (0.164–1.778). In univariable analysis in CMV seropositive KTx recipients (n = 256, 23 HZ cases), use of CMV prophylaxis significantly reduced the incidence of HZ (p = 0.0001, RR = 0.109, CI = 0.032–0.371).

## Discussion

Our study is one of the largest European studies that report the incidence and severity of HZ in recipients of four solid organ transplants with a maximum follow-up time of 14 years in HTx, 12 years in LuTx, 6 years in LiTx, and 10 years in KTx recipients. In addition, risk factors for the development of HZ were analyzed.

The crude HZ incidence was 17.1% in the heart, 14.0% in the lung, 5.8% in the liver, and 9.2% in the kidney transplant recipients. The overall HZ incidence in HTx (30.7 cases/1,000 PY) and LuTx (38.8 cases/1,000 PY) recipients was significantly higher compared to LiTx (22.7 cases/1,000 PY) and KTx (14.5 cases/1,000 PY) recipients.

Overall and crude HZ incidence rates in our KTx group are lower than the reports of KTx recipients from Canada and the USA, but higher than those from other European countries (14–16, 20, 22–25). HZ incidence in our LiTx group is higher than in previous studies (15, 19). These differences may be explained by a higher percentage of patients in North-America using T-cell depleting induction therapy and a more intense maintenance immunosuppressive regimen. In addition, in other European and Asian countries, the duration of CMV prophylaxis in KTx and LiTx patients is longer compared to our center. In our HTx and LuTx groups, HZ incidence is lower than in other studies (15, 17, 18, 21), which might be due to a longer duration of CMV prophylaxis in our transplant recipients and lower tacrolimus target trough levels after 1 year post-transplantation.

HTx, LuTx, and LiTx patients with infectious problems generally visit the outpatient transplant clinic. However, KTx recipients may visit their general practitioner and may go unnoticed. Therefore, KTx recipients wer also contacted by letter and by telephone. This resulted in a more reliable incidence and a more complete picture of HZ complications (*e.g.* PHN) compared to earlier studies that lacked this approach.

In addition to crude and overall incidences of HZ, our study also focused on severity of HZ. Post-herpetic neuralgia (PHN) and other complications of HZ are not uniformly described in the literature. In our study, PHN, dissemination, and cranial nerve involvement were more often reported in HTx and LuTx compared to KTx recipients. PHN incidence, as indicated in **Table 3**, is lower than in other reports. This is probably due to our more strict definition of PHN: >3 months PHN plus requirement of either opioid analgesics, tricyclic antidepressants, gabapentin, or pregabalin. The incidence of disseminated HZ was higher compared to other reports (14–19, 23). However, the definition of dissemination was not specified in these reports.

Furthermore, we analyzed potential risk factors for HZ. In the multivariate analysis, type of organ transplant, age ≥50 years at transplantation, duration of CMV prophylaxis, and type of anti-rejection therapy significantly influenced the risk of developing HZ (**Table 5**). As expected, the risk of HZ is higher in HTx and LuTx recipients, who are exposed to higher levels of immunosuppressive maintenance therapy. However, HZ incidence after KTx or LiTx is still significantly higher compared to healthy individuals (age <40 years: two cases/1,000 PY, age 40–50 years: 1–4/1,000 PY, age 50–70: 7–8/1,000 PY and age >80 years: 10/1,000 PY) (3). VZV and CMV are both herpes viruses; therefore CMV prophylaxis strategies might influence the incidence of HZ. Ko et al. found a lower HZ incidence per 1,000 PY in KTx patients who received >3 months prophylaxis compared to patients who received pre-emptive therapy (20). However, Fernandez-Ruiz et al. and Martin-Gandul et al. did not show a significant effect of CMV prophylaxis on HZ incidence compared to pre-emptive therapy (23, 25). In our study, patients using CMV prophylaxis did have a significantly lower risk of HZ. We did not find a significant effect of longer use of CMV prophylaxis on HZ incidence, but the number of patients receiving at least 6 months of prophylaxis (84 patients) may be too low to find significant effects. In our center herpes simplex prophylaxis with acyclovir is not used.

In other studies, more intensive immunosuppressive therapy was frequently reported as risk factor for HZ: anti-rejection treatment (21, 24), induction therapy (mostly anti-thymocyte globulin) (16), and use of mycophenolate mofetil (17, 19). Surprisingly, we found that in patients treated for acute rejection with methylprednisolone, the risk of HZ was lower than in patients who did not experience acute rejection. Sixteen out of 246 patients with methylprednisolone treated rejection developed HZ. We should not make assumptions due to these low numbers. However, our finding could be explained by the fact that most (overall 66%) of the methylprednisolone treated rejections occurred during the standard CMV prophylaxis period. Methylprednisolone could have diminished acute neuritis, although studies on steroid treatment of herpetic neuralgia show conflicting results (29, 30). Valganciclovir could have suppressed zoster virus reactivation in an early stage. Asymptomatic virus reactivations would then go unnoticed, but could have boosted the immune system. Another possible explanation is that patients experiencing rejection received an insufficient amount of immunosuppression and were therefore less immunocompromised compared to patients who did not experience rejection. More potent anti-rejection therapy with r-ATG was not a significant risk factor, possibly due to the low number of patients (eight of 48 with r-ATG developed HZ). Induction therapy no longer remained significant as risk factor for HZ after multivariable analysis. In our study, type of organ transplant appeared the most powerful risk factor for HZ. The higher level of maintenance immunosuppressive medication (higher tacrolimus target trough levels) is a probable explanation. However, type of organ transplant could also reflect more than the difference in immunosuppressive burden, but a more detailed analysis of patient’s frailty and co-morbidity is needed to confirm that hypothesis.

There are some limitations to our study. First, the patients included in the KTx and LiTx groups received their transplantation in different time periods compared to the HTx and LuTx groups. However, maintenance immunosuppression and CMV prophylaxis were comparable in all groups. Only induction therapy differed in the KTx group. Data on dosing of maintenance immunosuppression per patient were not analyzed due to the retrospective approach and large number of patients in this study. However, the target trough levels in each organ group are described in **Table 2**. Finally, we did not analyze renal function after solid organ transplantation as risk factor for HZ. Patients with end stage renal disease show premature aging of the T-cell system (31), and a higher HZ incidence in patients with chronic renal insufficiency has been reported (32, 33).

Vaccination has been shown to be effective in the prevention of HZ in healthy elderly people (34–36) as well as in patients with chronic kidney disease (37, 38). Currently, there are two licensed HZ vaccines. One is a live attenuated vaccine (34), which cannot be given to patients using immunosuppressive medication for fear of inducing VZV infection. The other is a subunit vaccine, containing VZV glycoprotein E (35), which could be given to patients on immunosuppressive medication, because it does not contain live virus. One phase III trial of the subunit vaccine in renal transplant recipients has shown persisting humoral and cell-mediated immunity at one year after vaccination (39). More studies are necessary to confirm that this vaccine is an effective tool to prevent HZ in organ transplant recipients.

In summary, this study shows that incidence of HZ is high after organ transplantation with severe complications. The incidence of HZ after HTx or LuTx is significantly higher than after LiTx or KTx. Use of CMV prophylaxis significantly decreases HZ incidence. A rational method to prevent HZ after organ transplantation might be to use CMV prophylaxis, and in our opinion booster vaccination in seropositive transplant candidates is advisable.

## Data Availability Statement

The data analyzed in this study is subject to the following licenses/restrictions: The data were derived from hospital charts and hospital pharmacy records. Requests to access these datasets should be directed to m.kho@erasmusmc.nl.

## Ethics Statement

The studies involving human participants were reviewed and approved by the Medisch Ethische Toetsings Commissie, Erasmus Medical Center, Rotterdam, Netherlands. Written informed consent for participation was not required for this study in accordance with the national legislation and the institutional requirements.

## Author Contributions

MK and NV designed the research, collected, analyzed, and interpreted the data, and prepared the manuscript. SR and DB participated in data collection. JR participated in statistical analysis and preparation of the manuscript. HM, RH, AV, and OM participated in interpretation and preparation of the manuscript. All authors contributed to the article and approved the submitted version.

## Conflict of Interest

The authors declare that the research was conducted in the absence of any commercial or financial relationships that could be construed as a potential conflict of interest.

## Abbreviations

ABO-I, ABO-blood type incompatible; CMV, cytomegalovirus; DCD, donation after cardiac death; HZ, herpes zoster; HTx, heart transplantation; LiTx, liver transplantation; LuTx, lung transplantation; KTx, kidney transplantation; PCR, polymerase chain reaction; PHN, post-herpetic neuralgia; PY, person years; r-ATG, rabbit anti-thymocyte globulin; SOT, solid organ transplantation; Tx, transplantation; VZV, varicella zoster virus.
